# Using soil survey data to model potential *Coccidioides* soil habitat and inform Valley fever epidemiology

**DOI:** 10.1371/journal.pone.0247263

**Published:** 2021-02-19

**Authors:** Robert R. Dobos, Kaitlin Benedict, Brendan R. Jackson, Orion Z. McCotter

**Affiliations:** 1 Soil and Plant Science Division, United States Department of Agriculture, Natural Resources Conservation Service, Washington, D.C., United States of America; 2 Centers for Disease Control and Prevention, Atlanta, Georgia, United States of America; University of Texas at San Antonio, UNITED STATES

## Abstract

Coccidioidomycosis, also known as Valley fever, is a disease that can result in substantial illness and death. It is most common in the southwestern United States and areas of Latin America with arid climates, though reports increasingly suggest its range is wider than previously recognized. The natural habitat of the causative organisms, *Coccidioides* spp., have been associated with certain soil properties and climatic conditions. Current understanding of its geographic range is primarily defined by skin test studies and outbreak locations. We developed a fuzzy system model to predict suitable soil habitats for *Coccidioides* across the western United States based on parameters (electrical conductivity, organic matter content, pH, water holding capacity, temperature, and precipitation) from sites where soil sampling has confirmed the presence of *Coccidioides*. The model identified high coccidioidomycosis incidence areas as having high suitability and identified pockets of elevated suitability corresponding with outbreak locations outside the traditional range. By providing high-resolution estimates of *Coccidioides* suitability, including areas without public health surveillance for coccidioidomycosis, this model may be able to aid public health and clinical provider decision making. Awareness of possible *Coccidioides* soil habitats could help mitigate risk during soil-disturbing activities and help providers improve coccidioidomycosis diagnosis and treatment.

## Introduction

Coccidioidomycosis (also called “Valley fever”) is caused by inhalation of airborne arthroconidia of the soil-borne fungi *Coccidioides immitis* and *Coccidioides posadasii*, which are endemic to the southwestern United States, northern Mexico, and parts of Central and South America. Because the symptoms of coccidioidomycosis, which include cough, fever, and fatigue, are similar to those of other respiratory infections, many patients experience delays in diagnosis and inappropriate treatment with antibacterial medications for presumed bacterial pneumonia.

The historical geographic distribution of *Coccidioides* roughly corresponds with the “Lower Sonoran Life Zone,” an area of similar plant and animal communities [1]. This distribution was estimated largely based on coccidioidin skin test reactivity [1–4]. In the United States, the first such comprehensive description involved coccidioidin skin tests of over 100,000 participants, including 50,000 who had spent their lifetime in a single county [4]. The study identified parts of Arizona, California, Nevada, New Mexico, Utah, and Texas as endemic, with the highest positivity rates (50%–70%) in California’s southern San Joaquin Valley, Arizona’s Sonoran Desert, and Texas’s Rio Grande Valley [4]. However, *Coccidioides* has recently been identified in northeastern Utah and south-central Washington State, which are distant from the traditionally defined endemic areas, indicating that its geographic range is likely wider than is currently appreciated [5,6]. Furthermore, *Coccidioides* distribution is patchy even within endemic areas [7], and currently available maps of estimated *Coccidioides* distribution across the western United States are based on rough estimations from human exposure rather than more spatially explicit models of soil and site properties.

Isolation of *Coccidioides* from soil and reports of locally acquired cases have identified endemic locations on the fringes of its traditionally defined range that also have hot summers, mild winters, and limited precipitation [8]. *Coccidioides* reportedly exists mainly in areas with mean annual air temperatures ranging from 15 to over 22 degrees Celsius [9]. However, soil temperatures likely have an even greater impact on presence of this fungus. Although air temperature and soil temperature are related, soil temperature is further affected by solar heating based on the slope, reflectance, and aspect of the surface, suggesting these features must also be considered [10,11].

Laboratory and site-specific field studies have also identified soil properties, such as salinity, pH, organic matter content, available water, and particle size distribution to be correlated with the distribution of *Coccidioides* [10,12,13]. Specifically, soils with *Coccidioides* often have high levels of soluble salts (salinity), likely because high salinity and high temperature reduce microbial competition [10,12,14,15]. The distribution of soluble salts in the soil is associated with water movement and evaporation, so landscape position affects salinity [16,17] and in turn, *Coccidioides* [18]. Regarding organic material, the soil-borne phase of *Coccidioides* is saprophytic and so must have an organic energy source, but soils of the U.S. southwest are notoriously low in organic matter, which is known to be unevenly distributed [10].

Soil moisture is also essential for the growth of *Coccidioides*, although precipitation in endemic areas is limited and seasonal. Southern Arizona and California’s Central Valley have important climatologic differences. California’s Central Valley is characterized by as a “xeric” soil moisture regime, with cool, moist winters and warm, dry summers [19], whereas Arizona has an “aridic” soil moisture regime, typified by high temperatures and modest, bimodal rainfall [19]. Yearly variation in rainfall and temperature can increase or decrease the distribution of *Coccidioides* [9]. *Coccidioides* has most commonly been detected in soils with higher proportion of sand than silt or clay, which have low water holding capacity compared soils with higher silt or clay content [9,10,20]. Departure from normal precipitation and soil moisture in previous years can influence disease incidence trends, with some studies suggesting that abundant *Coccidioides* growth occurs during high soil moisture periods followed by drying and airborne dispersal during subsequent arid periods, in a phenomenon known as “grow-and-blow” [13,21,22]. *Coccidioides* can remain dormant for long periods during persistent arid conditions and grow when the weather is suitable [10].

Previous environmental modeling studies have provided a strong foundation for understanding how precipitation, temperature, and soil properties influence the distribution of *Coccidioides* [23–26]. However, these studies primarily involve broad spatial resolutions or focus on relatively narrow geographic areas. A more comprehensive model of *Coccidioides* distribution in the United States could supplement information from prior environmental studies, historical skin test studies, outbreaks, and ongoing public health surveillance to improve understanding of the geographic risk of coccidioidomycosis. Additionally, a model covering large geographic areas with fine spatial resolution could help healthcare providers and public health professionals communicate about more specific areas and populations at risk, to reduce illness and delayed diagnosis. This model could also provide testable hypotheses about where this organism is more and less likely to be found. We therefore aimed to build a robust model based on multiple environmental parameters, including fine-scale soil data.

## Methods

### Soil survey databases

Our habitat suitability model uses data from 4 databases: the National Soil Information System (NASIS), the Soil Survey Geographic Database (SSURGO), gridded SSURGO, and the State Soil Geographic Database (STATSGO). Soil survey data for the United States are stored and continually updated in the transactional NASIS database, which is not publicly available [27]. The SSURGO database is publicly available and contains spatial and tabular data for each soil survey area, generally counties. Certain areas, like national forests, national parks, military bases, or some tribal lands, are unpublished because they currently lack any available soil survey data. The valid spatial scales for SSURGO range from 1:24,000 to 1:12,000 and have minimum delineation sizes of about 4 or 0.6 hectares, respectively. The tabular data capture estimates of ~300 attributes for each soil component in a survey area, its horizons, and the site characteristics of where it is found [28]. Site features include slope gradient, slope aspect, and slope shape, along with climate features such as mean annual precipitation and air temperature. Soil horizon data includes texture (sand, silt, and clay content), pH, available water, electrical conductivity, and cation exchange capacity, along with many other attributes [27]. The Gridded SSURGO (gSSURGO) database is a rasterized version of the vector SSURGO spatial data. The advantage of the rasterized spatial data in this study is that large (multistate or regions) areas can be rapidly displayed in GIS.

### Site characteristics and values

The model addresses chemical and physical soil properties along with climate and landscape variables. The suitability optimal ranges for each soil, site, and climatic property were gleaned from published literature whenever possible but do not have well-defined boundaries and are often highly related. The soil survey database reports mean annual air temperature and precipitation for each soil, so we used these simple parameters, with different ranges corresponding to ideal, possible, and not suitable habitat by xeric and non-xeric areas (Table 1). The mean annual precipitation (~230 mm) and air temperature (~20 degrees C) in the Lower Sonoran Life Zone were used as the optima for habitat, but the model also accounts for more xeric areas where the precipitation is higher (~400 mm/year) and air temperature is lower (~15 degrees C). Further refinement of the temperature and precipitation optima were obtained by intersecting climate data with mapped endemic areas [9,24,25]. To account for the well-characterized effects of local soil temperature heating, the model also includes southerly slope aspect, moderate slope gradient, and low surface reflectance (albedo). These microclimate effects are most important when the local mean annual air temperature is low. Although precipitation in these arid regions is sparse, moisture must be present in the soil for the saprophytic phase to grow. As water holding capacity and soil texture are strongly related, we used available water holding capacity since it more strongly influences soil-borne organisms.

**Table 1:** Thresholds and optima for soil and climatic properties.

Soil or Site Property	Not Suitable Habitat	Possible Habitat	Ideal Habitat
Electrical Conductivity* (dS/m, 0–30 cm depth)	<0.5	0.5 to 10	>10
Sodium Absorption Ratio (0–30 cm depth)	<10	10 to 50	>50
Gypsum Content (percent 0–30 cm depth)	<2	2 to 15	>15
Soil Reaction (pH) (0–30 cm depth)	<8	8 to 9	>9
Mean Annual Precipitation (xeric) (mm)	Below 50	50 to 400	400
	Above 800	400 to 800	
Mean Annual Precipitation (non-xeric) (mm)	Below 30	30 to 190	190
	Above 800	190 to 800	
Mean Annual Air Temperature (xeric) (C)	Below 10	10 to 15	15–18
	Above 30	15 to 30	
Mean Annual Air Temperature (non-xeric) (C)	Below 12	12 to 17	17–20
	Above 30	17 to 30	
Slope Aspect (degrees)		0–80	80–280
		280–360	
Slope gradient (percent)		0–8	8–15
		>15	
Albedo		0.1–1	<0.1
Land Surface Shape		Linear	Concave
		Convex	
Near Surface Anaerobic Conditions			
1) Number of Months with Ponding	> = 10	9 to 2	<1
2) Number of Months with Water within 30cm of the surface	> = 10	9 to 2	<1
Organic Carbon Content (kg/m^2^) (0 to 30 cm depth)		<3	> = 3
Water Retention Difference (cm/cm) (weighted average, 0 to 30 cm depth)	<2	2 to 10	>10

* EC, in decisiemens per meter (dS m-1) of an extract from saturated soil paste.

We approximated salinity from electrical conductivity (EC) in units of decisiemens per meter (dS/m). Optima and ranges for EC were inferred from laboratory studies [12,15] and from the reported EC in soils where the presence of *Coccidioides* has been confirmed [10,14,20]. Additional indicators of soluble salts include pH ≥8.0, greater presence of gypsum, and higher sodium absorption ratio (SAR). The thresholds for these additional chemical parameters were based on their correlation with EC as a means of ensuring as many soils having soluble salts as possible would be included in the model. Slope aspect (the direction the slope faces) and gradient thresholds were set to capture the solar heating of a south-facing land surface of moderate slope gradient and low reflectance (albedo). These parameters are intended to capture warm microclimates on the climatic suitability fringe and are not meant to exclude soils in the typical climatic regions. The shape of the land surface influences water flow through the soil and the movement of materials across the land surface, so we also considered surface morphology. Concave areas of the landscape, such as along streams, are noted as places that are favorable for the growth of *C*. *immitis* [18]. The near surface saturation criteria are based on the fact that the fungi are aerobic and only short duration of saturation or ponding will be tolerated. The amount of time required for the soil to dry out after being wet is taken into account by setting the threshold at no more than two months of saturation. Optimally, no ponding or saturation should occur. Organic carbon content thresholds and optima are based on the data provided in SSURGO for soil series reported in the literature [14,20] and what is typical for the Lower Sonoran Life Zone. A soil will not be classed as unsuitable for habitat based on low (less than 3 kg per square meter) organic carbon content. The water holding capacity of soil has been noted as a possible factor in *Coccidioides* growth [10,14,20] so water retention difference, which is not affected by salinity effects, is included in the model. The lower threshold of 2 cm/cm represents the water holding capacity of a very sandy soil and the optimum of 10 cm/cm is typical of a loam. *Coccidioides* spp. has typically been isolated from soil at depths of 2 to 20 cm; therefore, we used data for EC, pH, SAR, water retention, gypsum, and organic matter for the upper 30 cm of the soil rather than including lower depths.

### Fuzzy model

A rule-based fuzzy logic model first quantifies the degree of membership individual variables have in the set of variables that influence an outcome and then combines the individual effects into an overall model [29]. Fuzzy sets quantify the ambiguity of classes of objects that do not have well-defined boundaries, which allows a way to handle ambiguity or uncertainty as a result of a lack of defined criteria rather than random error [30,31]. Thus, the thresholds, as listed above, are expected to have some imprecision, and may vary spatially. Each soil property in a fuzzy set has a degree of membership, ranging from 0 to 1, which is established by a membership graph of the quantity of the property being evaluated versus its impact, in this case, its effect on the soil habitat for *Coccidioides*. For example, the membership graph of surface electrical conductivity (EC) might demonstrate that an EC of 1 dS/m is a non-member of the set of soils suitable for the fungus and provides a membership value of 0.0, EC 5 dS/m is a partial member with a membership value of 0.7, and EC 10 dS/m is a full member with a membership value of 1.0 [31]. We used the NASIS soil interpretations generator to develop a membership graph for each attribute we considered. This software uses a rule-based fuzzy logic system to model the degree of similarity of soil, landscape, and climate characteristics of all areas in the western United States with available SSURGO data compared to parameters estimated to be most suitable for *Coccidioides*. The NASIS soil interpretations generator is proprietary software designed to function within the overall soil survey database system. A complete description of the model can be found at https://dx.doi.org/10.17504/protocols.io.bh6gj9bw.

### Habitat suitability index

We modeled the presence and degree of similarity of soil and climate conditions similar to highly endemic areas where *Coccidioides* has been identified using the NASIS database’s fuzzy system module [32]. Finalized fiscal year 2018 data were exported from NASIS [32] and mapped using gridded SSURGO [33] in ArcGIS Desktop 10.5 (Esri, 2016). The actual data used in the model can be found at https://dx.doi.org/10.17504/protocols.io.bibakaie. To map the output, the data are map unit aggregated. We mapped the highest index of the major soil components if more than one existed. The attribute data used to make the maps can be found at https://dx.doi.org/10.17504/protocols.io.bia7kahn.

The habitat suitability index (HSI) ranges from 0 to 1, where 1 represents the combination of habitat parameters thought to be the most suitable for *Coccidioides*, and 0 indicates the site is least suitable for *Coccidioides*. The index is calculated by finding the square root of product of the membership values of the soil, climate, and site properties. We defined the HSI ranges of 1.0–0.8 as highly suitable, 0.8–0.5 as suitable, 0.2–0.5 as moderately suitable, 0.1–0.2 as somewhat suitable, and 0.1–0 as not suitable. The scale of the resulting map is in the range of the soil survey (i.e., 1:24,000 to 1:12,000), which allows possible habitat areas as small as a few hectares to be identified. We then compared results of the HSI with several data sources to evaluate performance: areas defined as endemic by skin testing, areas with elevated incidence of reported coccidioidomycosis, and areas with recent soil testing for *Coccidioides*.

## Results

**Fig 1:**
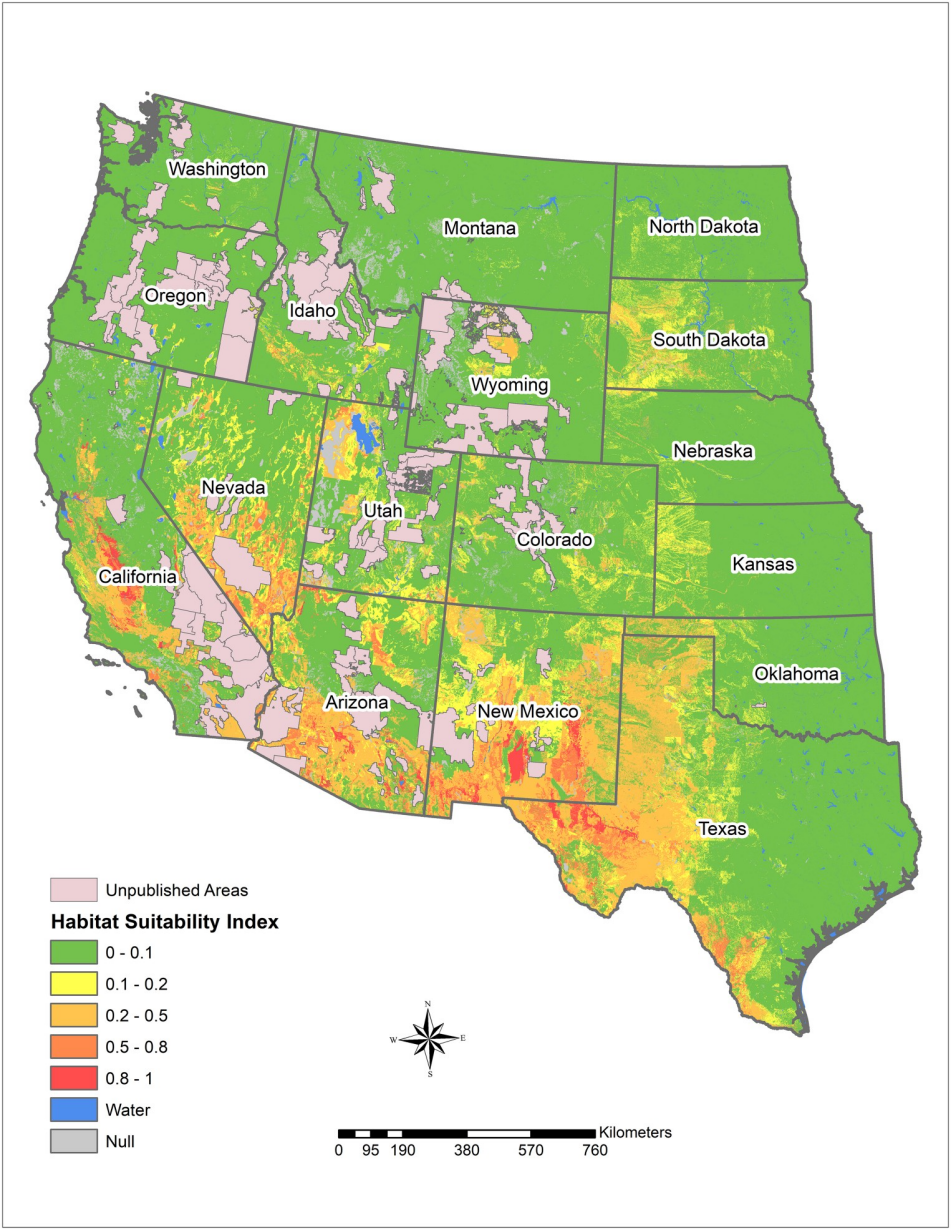
Predicted *Coccidioides* habitat suitability index for the western United States.

The HSI results show overall agreement with the areas known to be highly endemic for *Coccidioides*. For example, large areas of the San Joaquin Valley in California and south-central Arizona near Phoenix are modeled to be suitable for *Coccidioides* (Fig 1). In these areas with high temperatures (thermic and hyperthermic areas), the predicted habitat is closely aligned with the soil salinity, since typical temperatures are high, and heating of the soil surface occurs at most slope aspects. Moving northward or eastward from the areas with highest HSI, environmental conditions are predicted to become generally less favorable for *Coccidioides* (though pockets of increased HSI exist far beyond the classical endemic region). Least suitable areas for *Coccidioides*, shaded in green, are generally too wet, too cold, or lack sufficient soil salinity to support its presence in the model. The model does not cover certain areas of the western United States, predominantly involving unpublished areas lacking publicly available soil survey data in NASIS and SSURGO (shaded in pink) but also because of insufficient available data to generate an HSI (shaded in gray).

**Fig 2:**
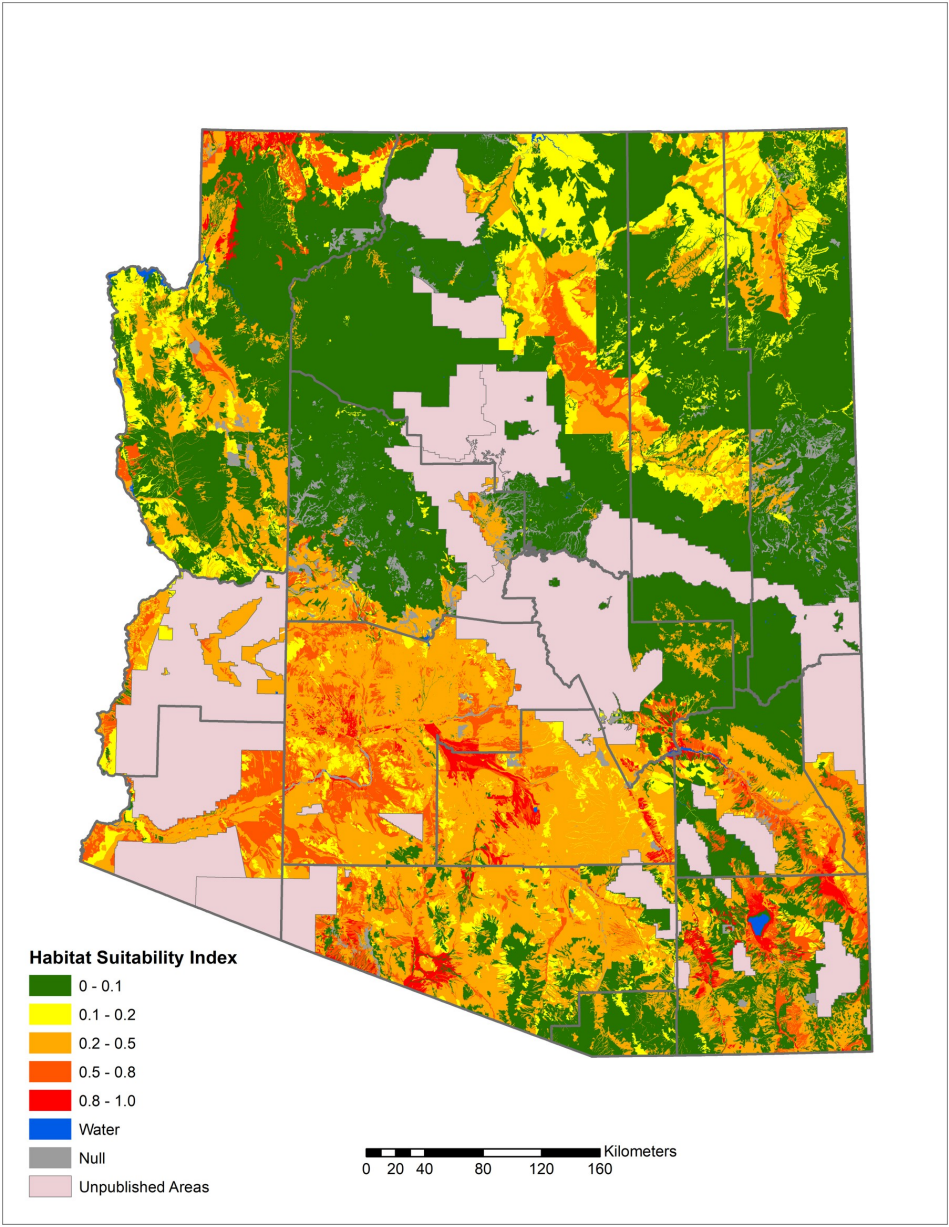
Predicted *Coccidioides* habitat suitability index for Arizona.

**Fig 3:**
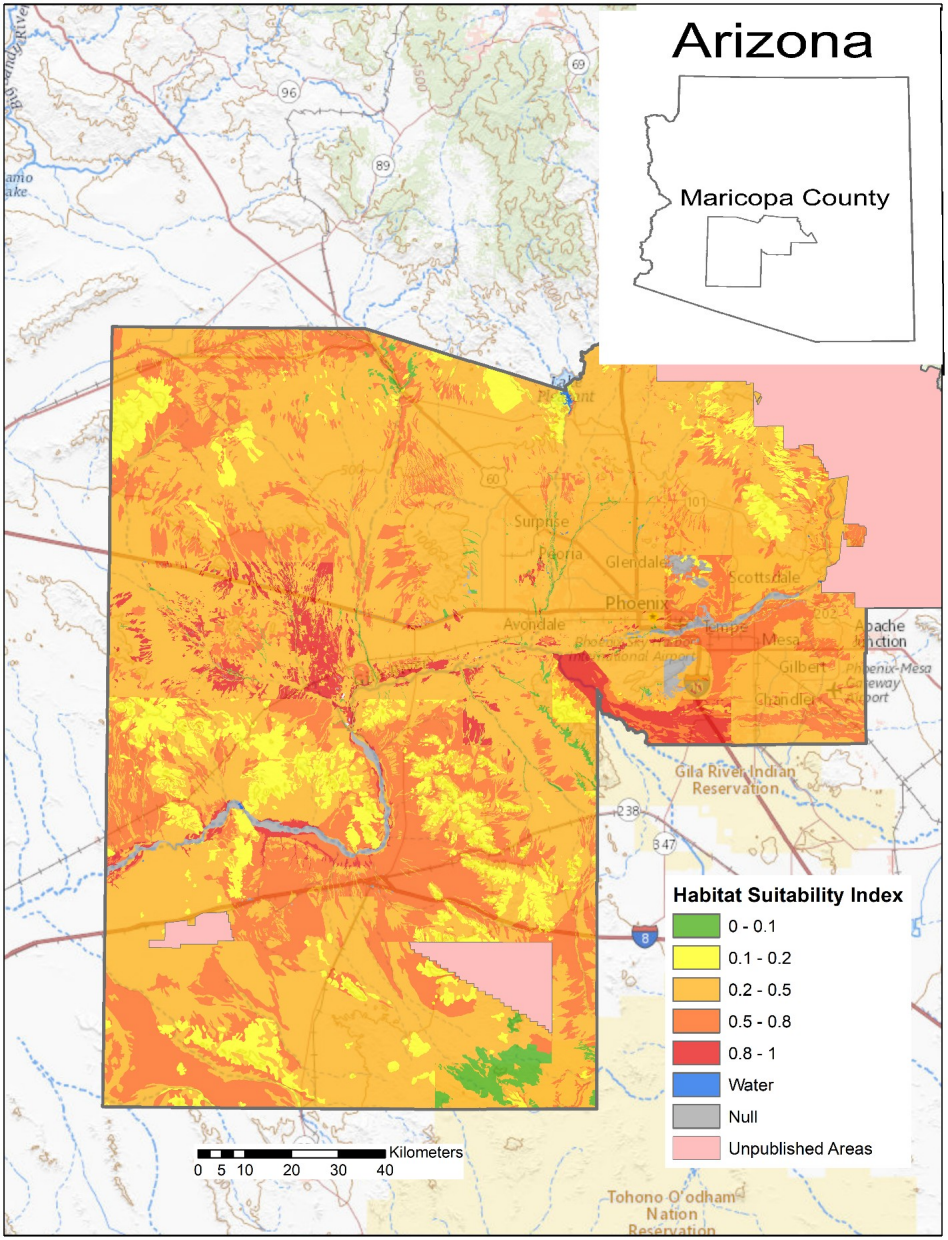
Predicted *Coccidioides* habitat suitability index for Maricopa County, Arizona, which has the highest incidence of coccidioidomycosis in the state.

**Fig 4:**
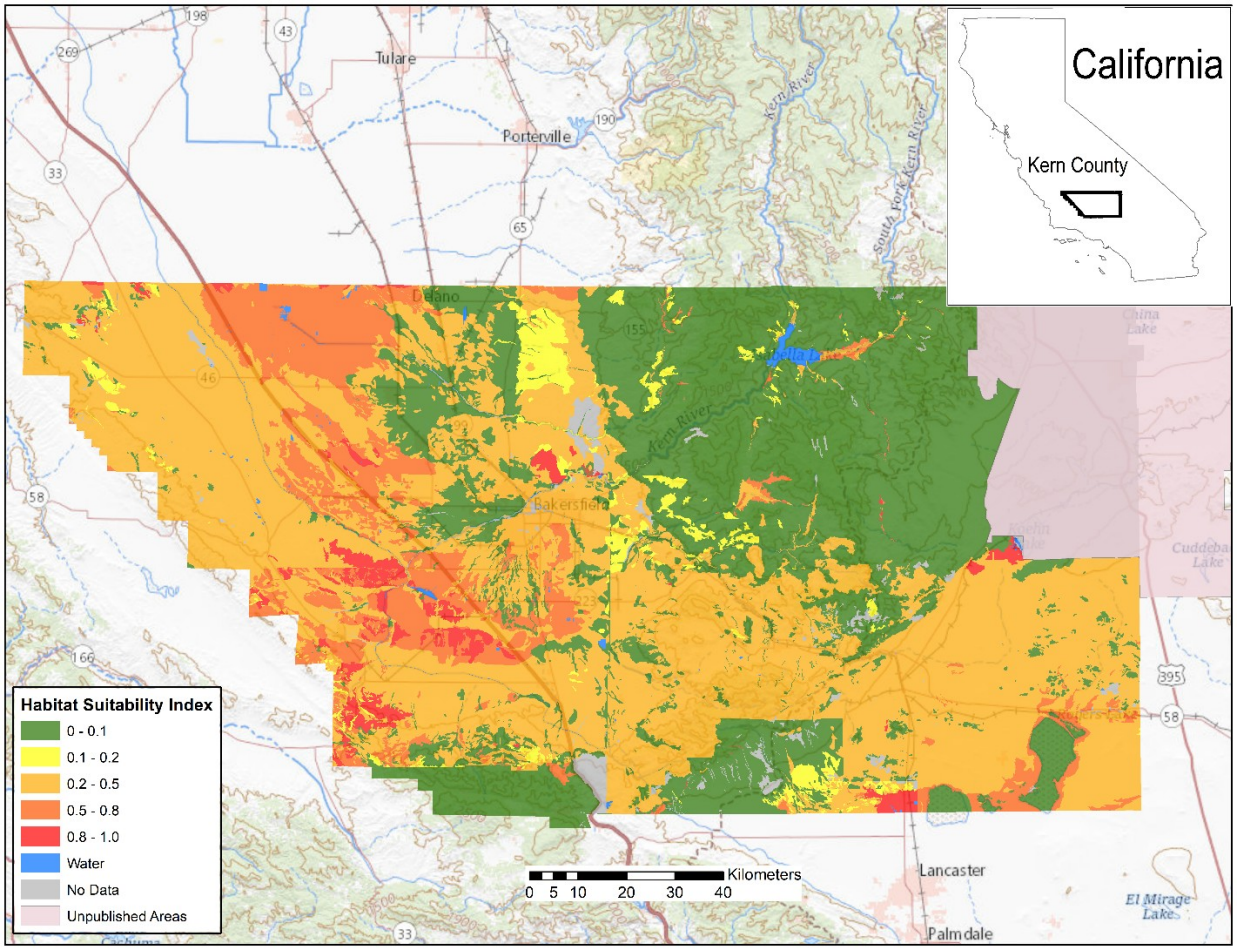
Predicted *Coccidioides* habitat suitability index for Kern County, California, which has the highest incidence of coccidioidomycosis in the state.

**Fig 5:**
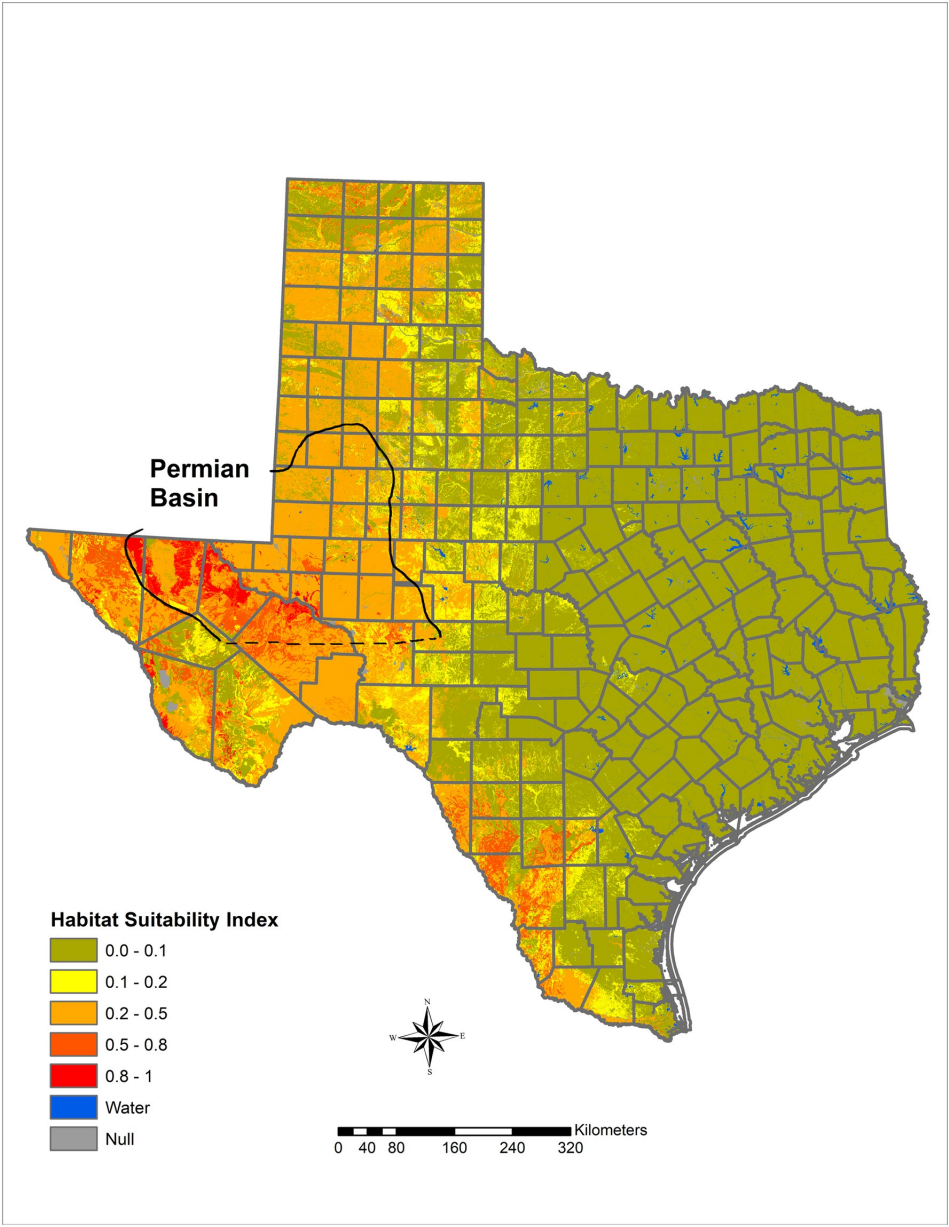
Predicted *Coccidioides* habitat suitability index for Texas.

**Fig 6:**
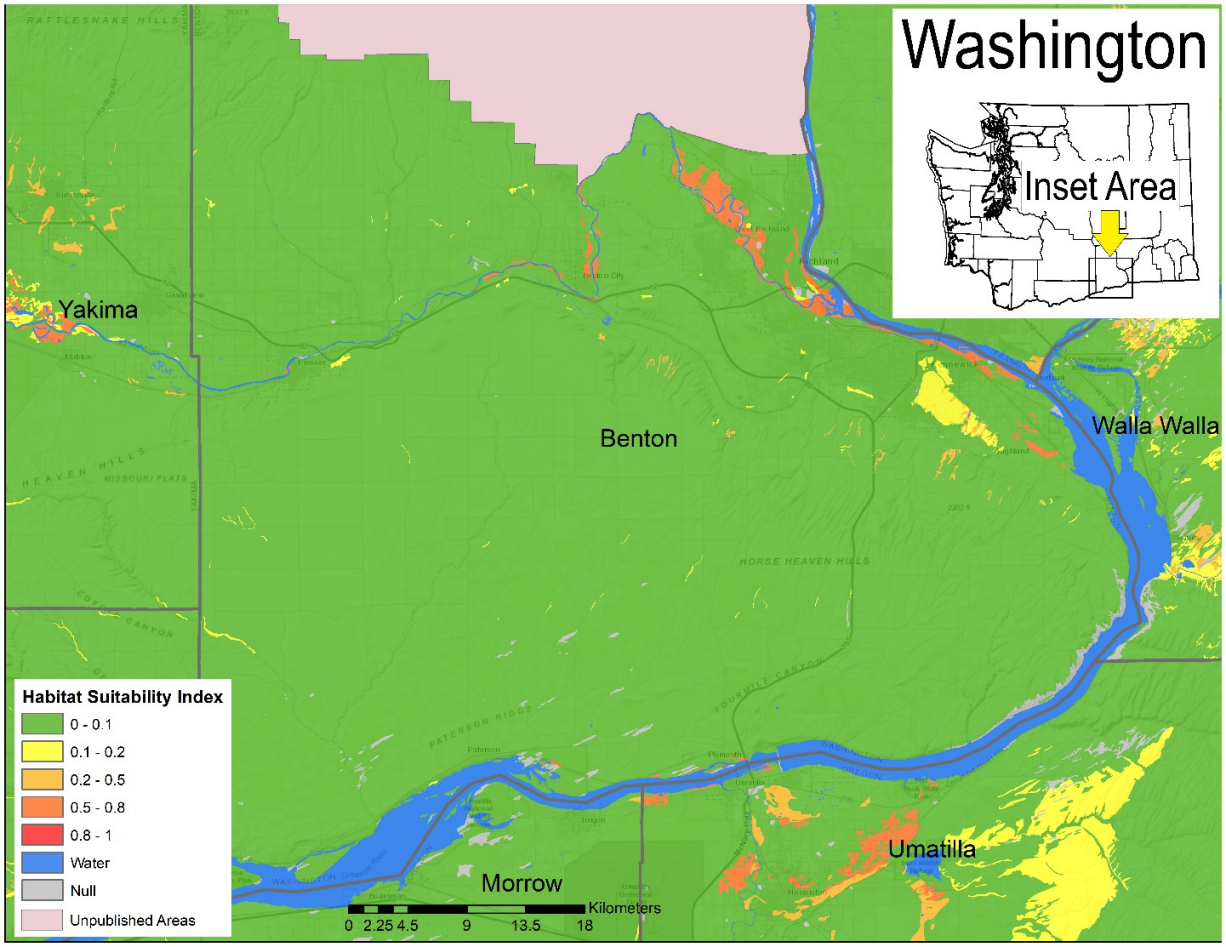
Predicted *Coccidioides* habitat suitability index for south-central Washington.

**Fig 7:**
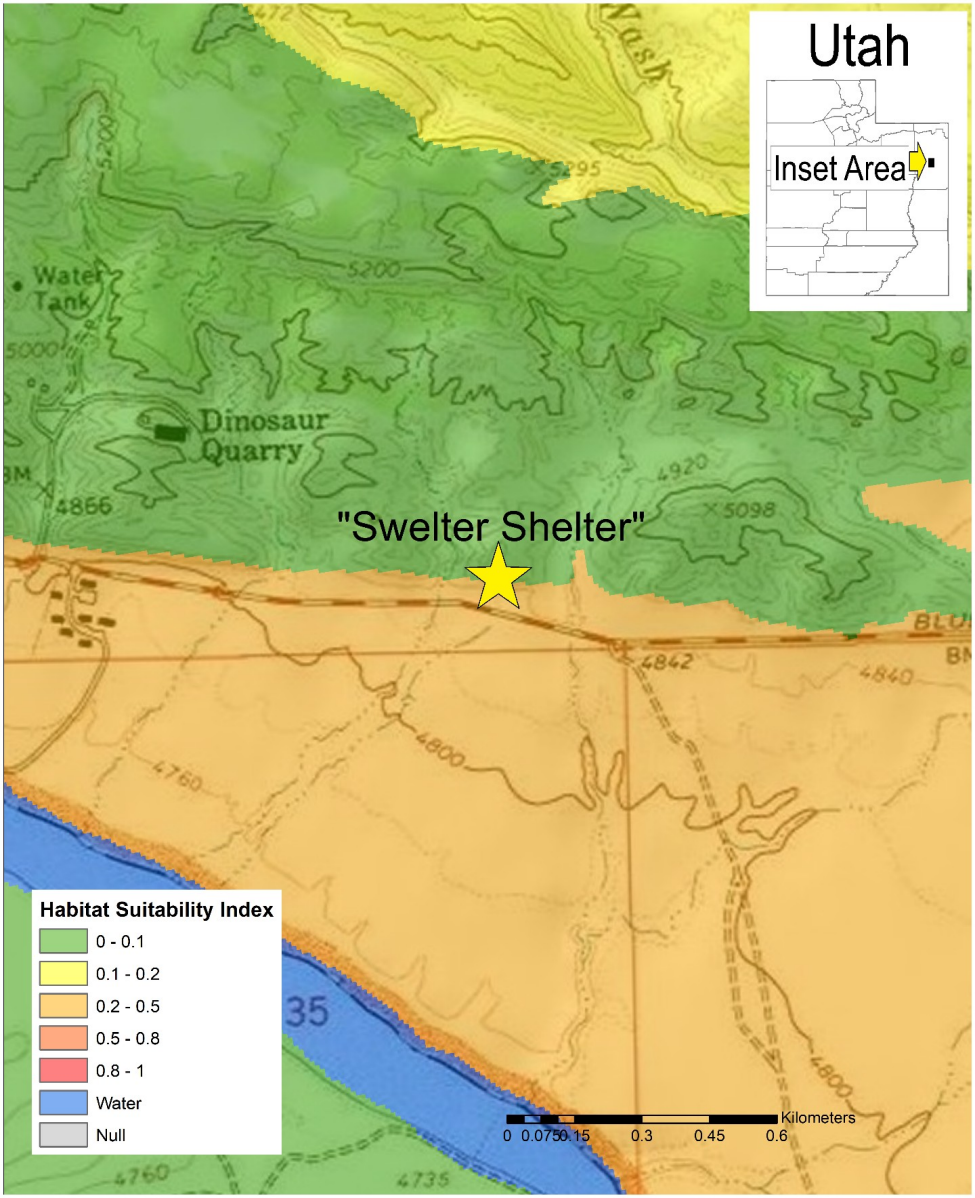
Predicted *Coccidioides* habitat suitability index for Dinosaur National Monument, Utah.

In Arizona, a relatively small proportion of the land area with publicly available data is highly suitable for *Coccidioides*, but a large portion of the southcentral area of the state is moderately suitable (Fig 2). Moderately suitable areas may represent those where certain weather conditions exist and where local variations in salinity, moisture content, and heat loading create hot spots of suitable habitat. Increasing the resolution to focus on Maricopa County, Arizona, which has some of the highest rates of coccidioidomycosis in the state, shows variable levels of suitable habitat, from sparse highly suitable areas and many areas with suitable to moderate ranges (Fig 3). Similarly, Kern County, the highest incidence county in California, has some areas with highly suitable habitat, and large areas of suitable habitat in the western side of the county. The remainder of the county is moderately suitable except for areas such as the high elevation areas in Sequoia National Forest (Fig 4). The model identified moderately suitable areas throughout the western portion of Texas and highly suitable in the Permian Basin. (Fig 5). In areas outside the traditionally described endemic regions, the model identified foci such as southcentral Washington State (Fig 6) and Dinosaur National Monument in northeastern Utah (Fig 7), both areas where locally acquired coccidioidomycosis has been detected.

We compared HSI output with results from *Coccidoides* soil testing in two studies in Southern California. In a study by Lauer *et al* [34], 13 of 24 sites tested in Kern County, California, had PCR testing suggestive of *Coccidioides* growth. Of the growth sites, HSIs were suitable for six, moderately suitable for five, and least suitable for two. Of the 11 sites without growth, four were suitable, 3 were moderately suitable, and 4 were least suitable. In another study, Colson *et al* detected *Coccidioides* at six of seven sites tested in the Antelope Valley area in Los Angeles County, California [35]; of the six sites with *Coccidioides* detection, one had a highly suitable HSI, one had a somewhat suitable HSI, and four had least suitable HSIs.

## Discussion

We developed a soil habitat suitability index to model and identify locations where *Coccidioides* may be more likely to exist across the western United States. Many of the areas with suitable soil habitats correspond with those historically considered to be endemic, which were defined by large-scale skin testing studies of humans and domesticated animals in the 1940s and 1950s. Replication of these skin testing studies are not feasible in today’s mobile population. This model provides an additional step towards enhancing our understanding of suitable habitats for *Coccidioides* spp., both by modeling the wide range of suitability present within historically defined endemic areas and by identifying potential pockets of increased suitability outside these areas.

Our model also correlates with case data from public health surveillance [36]. We identified large areas with highly suitable habitats in south-central Arizona and California’s southern San Joaquin Valley, which consistently have the highest incidence of reported coccidioidomycosis cases in the United States (S1 Fig) [37]. Within the San Joaquin Valley, Kern County has the highest incidence of reported coccidioidomycosis, and most cases are reported from the western half of the county [38], which is consistent with the areas of highest modeled suitability on finer resolution. Areas such as southern New Mexico are also part of the historically endemic areas and show similar habitat suitability, but have lower levels of reported disease, which could be related to low population density. This model also represents an additional method to better define the geographic risk in Texas, where the distribution of coccidioidomycosis is largely unknown, as it is not a reportable disease, apart from local surveillance in the city of El Paso. In Texas, the historic skin test study identified a high proportion of positive skin test reactions in areas from the Permian Basin through the Rio Grande Valley [4], both areas with high *Coccidioides* HSI. A review of canine serologic testing identified similar areas of rates of positivity [39]. More recently, a review from a large regional referral medical center in the west Texas city of Odessa reported substantial numbers of coccidioidomycosis diagnoses [40]. Accordingly, our model identified this area of the Permian Basin as having a highly suitable (0.8–1) HSI.

Our findings are also similar to previous ecologic niche modeling conducted at broader spatial resolutions. For example, our HSI generally supports a recent ecologic niche modeling study of *Coccidioides* using climate, soil, and land cover data to map a relative occurrence rate for the western United States [41]. They are also similar at the regional scale to those from a model by Baptista-Rosas *et al* that used environmental and epidemiologic data to predict hotspots of *Coccidioides* in the Southwest [23].

Understanding of geographical risk based on cases of coccidioidomycosis is challenging because precise exposure locations are seldom known. However, point source outbreaks and direct environmental detection allow for greater specificity to the exact locations of *Coccidioides*. Our model not only depicts places where the disease exists based on epidemiologic data, but also shows areas where *Coccidioides* historically have been isolated from soil and also where DNA has been detected with newly developed molecular techniques [8,14,42]. However, the model yielded imperfect alignment with recent soil detection of *Coccidioides* at specific sites in Southern California [34,35], suggesting future refinement of the model is needed. Soil conditions in SSURGO data are based on specific sampling sites within each 0.6 or 4 hectare area; because soil conditions can change within a matter of meters, even this relatively fine-scale model cannot account for all microhabitats, which may create some misalignment with soil microbiologic testing. Outbreaks of coccidioidomycosis that are linked to a common source are rare but also provide greater granularity of locations the fungus exists in the environment. Most known outbreaks have occurred in places with highly suitable habitat, and with this model even outlying areas can be examined at closer scale [43].

This model also examines areas at a finer resolution than has previously been attempted. For example, suitable habitats are predicted to exist in outlying locations, such as Washington State and northeastern Utah, areas that were generally considered unsuited for *Coccidioides*. In south-central Washington, suspicion of locally acquired cases began when a cluster of three coccidioidomycosis cases in people without recent travel to known endemic areas were identified [5]. This was later confirmed when *Coccidioides immitis* was cultured from the soil where one patient was exposed, with this isolate being genetically indistinguishable from a clinical isolate [44]. The model also predicts suitable habitat in areas of nearby north-central Oregon, although locally acquired cases have not been detected there. A coccidioidomycosis outbreak associated with the “Swelter Shelter” archeological middens excavation in Dinosaur National Monument provided the first evidence of the fungus in northeastern Utah [45], which was strengthened when *Coccidioides* DNA was identified there years later [46]. Our model predicts a moderately suitable habitat in this area, indicating that the local microclimate likely creates pockets of conditions favorable to *Coccidioides*. Temporal aspects related to yearly variations in rainfall and temperature likely also play a role.

Our model also predicts small suitable areas in Nebraska, Idaho, Wyoming, and South Dakota at the extreme edges of the known environmental range, where locally acquired coccidioidomycosis has not been identified in humans. Findings such as the identification of *Coccidioides* spherules in an early Holocene (8,500 years ago) period bison fossil from Nebraska could support this potential range [47]; however, the absence of more recent evidence of *Coccidioides* in this region suggests model outputs for this area must be interpreted with caution. Still, locations with predicted suitable habitats on the eastern edges of the known geographic range might warrant further investigation and a heightened suspicion for coccidioidomycosis. The unpublished areas joining suitable areas should also be further explored because many of those lands are used for recreational activities. Further work to test in soils in areas with high predicted HSI can be used to validate and improve this model.

A major limitation of this study is that it only accounts for suitable soil habitat for *Coccidioides* and does not account for soil disruption, dispersal of arthrospores, and resulting exposures to humans and animals. In general, infections occur in areas where *Coccidioides* grows in the environment. However, large-scale disturbances like the 1994 Northridge, California, earthquake, can cause spores to become airborne and produce outbreaks outside the immediately affected areas [48]. In rare cases, spores can also transmitted via fomites such as clothing or vehicles, causing cases in unexpected places [49]. Our model also does not address the potential role that small mammal carcasses might play in the distribution of *Coccidioides* [50]. Soils containing substantial amounts of organic matter, such as animal burrows or human middens, have been shown to be favorable to growth of *Coccidioides* spp. [51].

This model could be expanded to examine temporal trends such as precipitation and temperature. Previous work has attempted to correlate precipitation with coccidioidomycosis incidence in subsequent years, but such modeling is challenging given delays in identification and diagnosis of cases and differing precipitation patterns in Arizona and California [13,22,52]. Additionally, climate change could render areas not currently warm enough to support *Coccidioides* more suitable, while at the same time rendering other areas too hot or dry for the fungus to survive.

This model predicts areas with suitable habitats for *Coccidioides* in the western United States. Greater awareness of these areas could help the public and certain industries take extra precautions to mitigate risk when conducting soil-disturbing activities and help remind healthcare providers to consider coccidioidomycosis in patients who live in or travel to these areas.

## Supporting information

S1 Fig Average incidence of reported coccidioidomycosis per 100,000 people, by county, 2011–2017. (PDF)
