# Supplementary figures and images for: Using soil survey data to model potential Coccidioides soil habitat and inform Valley fever epidemiology
Source: PLoS One. 2021 Feb 19;16(2):e0247263. doi: 10.1371/journal.pone.0247263 (PMC7894876; doi:10.1371/journal.pone.0247263)

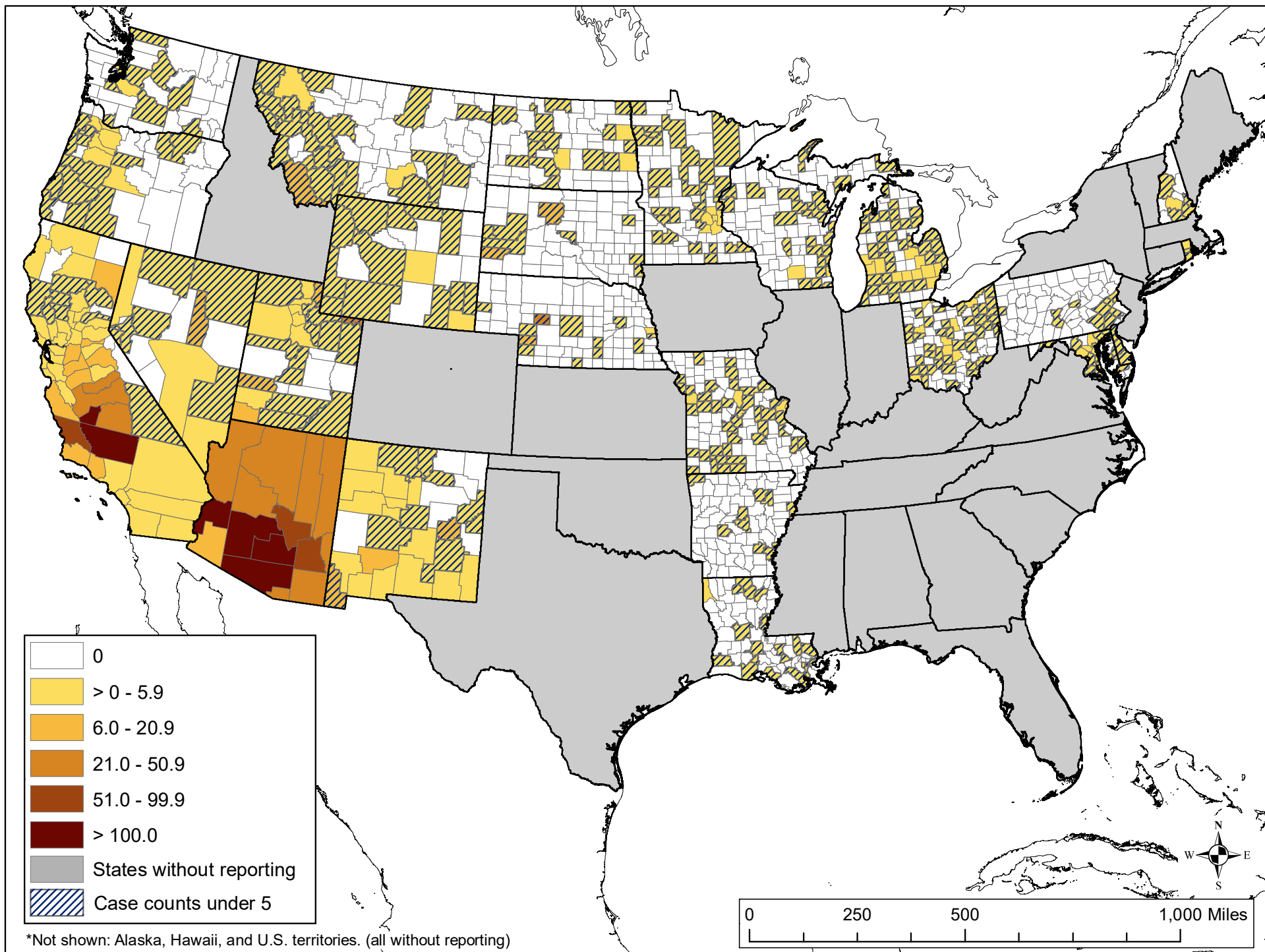

Supplement: S1 Fig — (PDF) [file pone.0247263.s001.pdf]
